# Case report: Malaria and hepatitis E coinfection—first experience with such imported entity in Serbia

**DOI:** 10.3389/fmed.2024.1498971

**Published:** 2024-12-03

**Authors:** Aleksandar Ranković, Maja Cvetanović, Jelena Miladinović, Milica Ćosić, Lidija Popović Dragonjić

**Affiliations:** ^1^Department of Infectious Diseases and Epidemiology, Faculty of Medicine, University of Niš, Niš, Serbia; ^2^Clinic for Infectology, University Clinical Center Niš, Niš, Serbia

**Keywords:** malaria, hepatitis E, imported disease, diagnosis, vaccine

## Abstract

Acute hepatitis E virus (HEV) and malaria are not rare infections in tropical countries; however, in the European continent, such imported entity has not been reported up to now. Herein, we report a 24-year-old male suffering from malaria and hepatitis E, who was admitted with acute hepatic failure dark colored urine, followed by coagulation and inflammation parameters increase. Blood smear analysis revealed the presence of *Plasmodium falciparum*, while serological tests revealed anti-HEV IgM antibodies. After the recommended antimalarial drugs and supportive therapy, the patient survived and was discharged disease-free. Conclusions: Following WHO guidelines for the treatment of severe malaria, full recovery in patient was achieved.

## 1 Introduction

Malaria is the most common tropical disease with high morbidity and mortality and an enormous consequent economic and social importance. It is estimated that 300–500 million people are affected annually, and around 1.5–3 million, mostly children, die from malaria every year (1). Approximately 30 thousand tourists from developed countries contract malaria during the year, and several hundred thousand people die from this disease (2). In the European Union, 11.000 cases of imported malaria are re-ported annually, and approximately 8.000 of these cases are malaria caused by Plasmodium falciparum (3). The World Health Organization confirmed that ‘Europe is free of malaria’ in 1974, while in former Yugoslavia, malaria was eradicated in 1964. Nevertheless, imported cases are registered in Serbia every year, and according to the data of the Institute for Public Health of Serbia, 159 cases of imported malaria were registered in the period 2010–2019 (4) and the period 2014–2018 according to the official data incidence rate of imported malaria ranged from 0.14–0.40/100,000 residents (5). Apart from malaria, other imported and previously eradicated diseases have emerged in Serbia and neighboring countries (6).

Malaria is a very complex disease that does not have any absolute clinical characteristics, and thus, it can easily mimic many diseases (7). The clinical presentation varies among individuals and depends on the level of malarial parasitemia and the immune status of the patient. The liver plays a vital role in the life cycle of the malaria parasite, and it can be severely affected in some cases. Jaundice is a common manifestation of a complicated falciparum infection. It is a result of intravascular hemolysis, hepatocellular dysfunction, or disseminated intravascular coagulopathy (DIC) (7, 8). The term ‘malarial hepatitis’ is often used to describe hepatocellular jaundice in patients with malarial infection (9, 10), and hepatic damage is often associated with severe malaria (11).

Hepatitis E virus (HEV) was previously considered as an insignificant member of Hepeviridae family, however, these days it is considered equally concerning as other hepatitis viruses. Although the virus is not new the clinicopathological features associated with hepatitis and limited resources for diagnosis (with still no standardized FDA-approved diagnostic tests) represent a challenge in managing it. The infection with HEV is frequent in immunocompromised patients as well as in pregnant women and its management can be challenging sometimes (12).

In endemic areas, it is important to remember that jaundice can be a part of malaria *per se*, or it can occur as a part of acute viral hepatitis. In patients with fever and jaundice with or without altered sensorium, disproportionate hyperbilirubinemia and only mildly elevated liver enzymes may help differentiate these cases of malaria from viral hepatitis (13). Acute hepatitis E and malaria are not rare infections in tropical countries, and they can coexist; however, in the European continent, such an entity has not been reported up to now. One case reports a fatal fulminant liver failure, with a combined infection with hepatitis E and falciparum malaria in a 20-year-old girl (14). A similar case of an elderly female patient was reported in India by Shaikh (15). Although their cases had fatal outcomes, a single case of complicated vivax malaria with coexisting acute hepatitis E had a positive outcome (16). A positive outcome of a severe form of cerebral malaria and hepatitis E infection in a 33-year-old patient treated in the USA after a stay in an endemic area of Africa was described recently (17).

In order to raise awareness about malaria in Serbia and even possible coinfection with the Hepatitis E virus, we report an unusual case of coinfection in a 24-year-old patient with ‘blackwater fever’ caused by Plasmodium spp.

### 1.1 Case report

**Figure 1:**
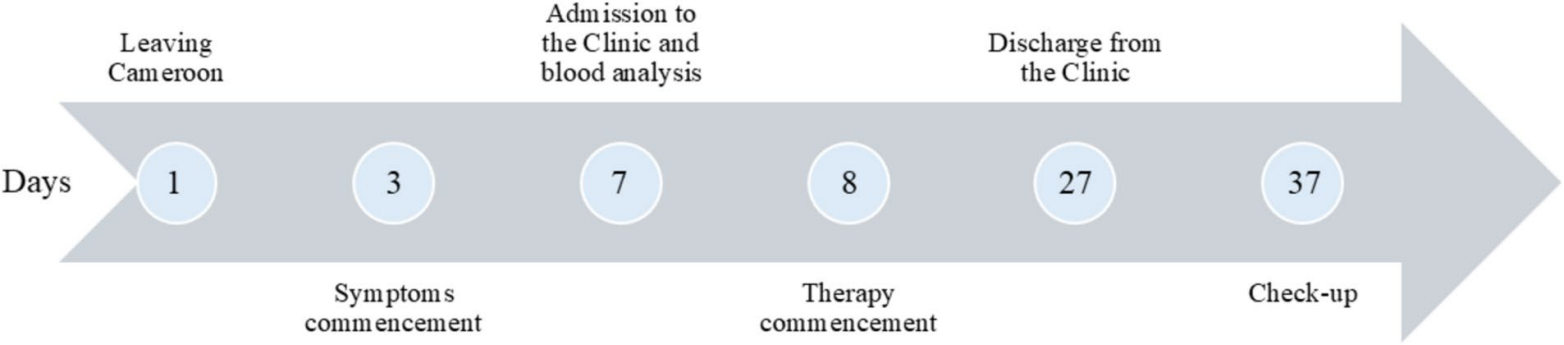
Timeline of the disease development and treatment.

A 24-year-old African male patient, born in Cameroon, currently living and working in Serbia, reported to the Infectious Diseases Clinic of the University Clinical Centre of Niš. Prior to admission, he was healthy and in good form. At the time of presentation, the patient complained of fever, headache, dizziness, abdominal pain, nausea without vomiting, liquid stools with traces of blood, as well as dysuria complaints (low dark-colored urine output). He has been experiencing the mentioned symptoms 4 days prior to the admission (Figure 1).

Epidemiologically, seven days before admission, he departed from the Republic of Cameroon for Serbia, where he had spent two weeks. During that period of time, he did not use any malaria prophylactic drugs even though the disease is endemic for this region and prophylactics are recommended. He mentions an episode of malaria in the past (3 years ago) and reports an allergy to quinine and quinine-based drugs. There is no other relevant data in his personal or family medical history.

While being admitted, during the physical examination, a high temperature (39°C/102.2°F), hypotension with a blood pressure of 100/60 mmHg, accompanied by tachycardia with a heart rate of 105 bpm, a breathing rate of 27 breaths per minute, and peripheral oxygen saturation of 96%, were registered. He was prostrated but with a normal state of consciousness. Also, mild sensitivity of the abdomen with a slightly enlarged liver and spleen was detected. There were no signs of cutaneous bleeding, and the examination revealed no neurological deficits.

Initial hematological tests showed that the patient has anemia (Hb - 108 g/L) and thrombocytopenia (PLT - 45x10ˆ9 /L). The liver function analysis were out of nor-mal/physiological range (total bilirubin – 73 μmol/L, direct bilirubin - 21.1 μmol/L, albumins - 27 g/L, ALT - 37 U/L, AST – 91 U/L, D-dimer - 14854 ng/mL); kidney tests indicated acute failure (urea - 9.1 mmol/L, creatinine - 143.6 μmol/L). Procalcitonin and C-reactive protein were markedly elevated (Table 1), indicating a septic condition.

**Table 1:** Results of laboratory tests during hospitalization and after the discharge.

Hematology	Day 1	Day 2	Day 4	Day 6	Day 9	Day 18	10 days after the discharge	Values
WBC (x10ˆ9/L)	5.6	9.0	19.2	15.6	15.3	7.4	6.7	4–9
Erythrocytes (x10ˆ12/L)	4.04	2.86	3.26	3.65	3.56	3.97	4.45	4.3–5.8
Hemoglobin (g/L)	108	83	83	94	96	112	122	120–180
PLT (x10ˆ9/L)	45	61	146	234	340	266	197	120–380
Urea (mmol/L)	9.1	9.2	7.5	6.6	4.9	5.2	5.6	2.5–7.5
Creatinine (μmol/L)	143.6	142.4	110.1	195	111.6	117.6	111	53–115
AST (U/L)	91	91	32	45	32	34	52	10–37
ALT (U/L)	37	31	27	50	38	44	25	10–42
Albumin (g/L)	27	25	25	27	30	37	-	35–52
Bilirubin (total) (μmol/L)	73	149.3	26.6	29.8	32.4	-	18.1	5–21.0
Bilirubin (direct) (μmol/L)	21.1	51.6	10.4	9	7.5	-	4.7	0–3.4
PCT (ng/mL)	89.88	61.14	12.69	2.9	1.3	0.28	-	0–0.05
CRP (mg/L)	404.8	209.9	122.6	55	16.1	10.3	1.6	0–5
LDH (U/L)	1,397	1823	1,144	839	935	610	358	220–450
Ferritin (μg/L)	1017.9	892.1	719.4	555.8	-	-	-	20–250
D-dimer (ng/mL)	14,854	943	-	593	342	210	-	0–250

Having in mind the travel history and the results of blood tests, a differential diagnosis of malaria was taken into consideration and a peripheral blood smear and a panel of biochemical tests for acute haptic failure were suggested. Also, a panel of immunological assays for viral hepatitis and other hepatotropic viruses was performed. A peripheral blood smear was positive for *P. falciparum*. The serum was positive for IgM HEV and negative for HBsAg, anti-HCV, anti-HIV, and leptospirosis. Taking into account the frequent and liquid stools, additional bacteriological, parasitological, and mycological examinations of the stool were performed, which all turned out to be negative. Urine analysis did not show any signs of urinary infection. Blood and urine culture tests turned out sterile. Ultrasonic examination of the abdomen showed that the liver and spleen were voluminous. A chest X-ray showed no abnormalities.

After confirming the diagnosis of malaria, a therapy based on the latest recommendations of the World Health Organization was given. Antimalarial therapy initially included intravenous artesunat (180 mg) during the first 24 h, afterward arthemether (160 mg per day) for six days, and finally artemether (80 mg) and Lumefantrine (480 mg) orally two times per day for three days. Antibiotic therapy in the form of ampullar clindamycin (600 mg/8 h) for 10 day and oral doxycycline (100 mg/12 h) for 7 days were administered in parallel to the artemisinin derivatives. Due to significant anemia and hypoalbuminemia, the patient was substituted with washed erythrocytes (350 mL on two occasions) and human albumin (50–100 mL) until these parameters were normalized. Also, supportive therapy in the form of saline solutions, and poly-vitamin were given daily.

After the therapy, a drastic improvement in the clinical findings and blood tests was noted. The liver enzymes were decreased, the number of platelets returned to normal, and the values of erythrocytes, hematocrit, and albumin started returning to normal values, as did the values of D-dimer (Table 1). Renal function was also recovered. Parasitemia disappeared, which was confirmed by three negative peripheral blood smears, and the patient was discharged from the hospital after 19 days. At the fol-low-up examination (10 days after being discharged), the patient was asymptomatic. Routine laboratory tests and urine analysis were within reference values.

## 2 Discussion

The diagnostics conducted on this patient represented a challenge to a certain extent since both malaria and HEV could cause liver damage and changes in blood biochemical parameters observed in patient. Since he returned from Africa and had a previous history of malaria, with presenting symptoms, the patient was immediately screened for blood parasitemia (without quantification). Also, since he was coming from an area with high frequency of hepatitis, including HEV (18), which is underreported, and poorly managed, the author’s idea was to screen the blood sample toward a panel of hepatitis viruses. After appropriate anamnestic data collection, the algorithm for both malaria and hepatitis was evident, however, a routine screening for blood and urine culture was performed in order to exclude other causes. The detection of HEV was done according to the standard procedure of the referent laboratory of the department of virology using both ELISA for HEV IgM and automated immuno-diagnostic system based on enhanced chemiluminescence. The detection of HEV was only based on serology due to the fact that he was coming from an endemic area (18) and in such cases the serological confirmation is deemed satisfactory (19). These algorithms are followed for all patients coming from endemic areas, such as Africa, and are recommended by the guidelines in order to discover imported diseases. This way the diagnosis of malaria and HEV might be more prompt than in non-endemic countries.

Malaria is an infectious disease with a high degree of mortality and the development of various complications. Acute intravascular hemolysis is one of the few complications of malaria that leads to blackwater fever. A review of the literature has de-scribed cases of glucose-6-phosphate dehydrogenase (G6PD) deficiency as well as irregularly taken quinine and other quinolone derivatives that are linked to the pathogenesis of blackwater fever (20). This clinical report describes a man allergic to quinine who was repeatedly treated for Plasmodium sp. dark-colored urine (black) was found immediately upon admission of our patient. The same feature was not present in patients reported for the malaria and HEV co-infection (14–17). Unfortunately, we were not able to deter-mine the level of G6PD since it is not done routinely in our laboratories. Whether previous quinine use or G6PD deficiency underlies the observed syndrome remains un-known.

The development of sepsis can accompany severe *P. falciparum* malaria as one of the many complications of the disease with a possibly fatal outcome. A recommendation of the World Health Organization for the treatment of malaria with the simultaneous use of broad-spectrum parenteral antibiotics with parenteral antimalarials, the treatment of this disease has become more accessible, with the proven positive effects (21). Combining doxycycline and clindamycin with parenteral artemisinin derivatives resulted in a positive treatment outcome and prevention of complications in our patient. In all reported cases the therapy included antimalarial drug artesunate, however, the outcome was not always favorable (14–17) as in the present case.

It is generally known that both acute kidney failure and the development of hemolytic uremic syndrome (HUS) can be severe complications of malaria (7). The mechanism of secondary HUS in malaria is not fully understood, but it is believed that the cascade activation of inflammatory cytokines leads precisely to the impairment of renal function (7). However, thrombocytopenia, anemia, and azotemia, along with an increase in LDH in our patient, resulted in the withdrawal of disease symptoms with a complete recovery of renal function due to the timely administration of antimalarials. Thus, a gradual improvement in the patient’s clinical and laboratory status correlated with the elimination of the parasite.

An aggravating circumstance of this patient’s presentation is his simultaneous infection with HEV. It is known that the prevalence of viral hepatitis E is high in African countries (22) and as mentioned in other report on this co-infection the patients returning from Africa and endemic areas for hepatitis should be tested with a panel of test for hepatitis (17). Hyperbilirubinemia, icteric sclerae, and his recent return from a trip to Africa prompted us to do acute hepatitis panel testing. Hepatitis E IgM (HEV IgM) was positive, while the rest of the hepatitis panel parameters turned out negative, which served sufficient for HEV diagnosis in previous report (14, 15, 17). The patient reported gastrointestinal symptoms in the form of nausea without vomiting. Slightly elevated values of transaminases were verified along with voluminous liver and spleen.

The management of HEV is slightly improved by the discovery of a HEV vaccine, currently only one register Hecolin, which is a recombinant vaccine effective against the most common genotypes (1 and 4). In endemic areas the vaccine would help prevent disease spread and could prevent infection in risky population (pregnant women and immunocompromised). In non-endemic regions the need for this vaccine is low and can be recommended to travelers, individuals working with animals, and certain healthcare workers.

Patient with imported infections has been examined and threated thoroughly at the Clinic of Infectious diseases, University Clinical Centre Niš. The state of the patient fluctuated from its admission when his life was in danger to complete recovery. Although he was adapted to living in Serbia serious infection and hospitalization must have greatly influenced his psychic state since he was alone in a foreign country, with significant health disturbances. Furthermore, foreign language, different ways of communication and interaction, with a sizable language barrier must have contributed patient state. This perspective should be taken into account when treating foreign patients.

## 3 Conclusion

Despite the low probability of clinicians and laboratory staff in our area encountering malaria, which is more common due to large population migrations from malaria endemic areas, timely diagnosis and therapy are important for patients coming from endemic areas. The coinfection with hepatitis E is even less probable but for patients coming from the endemic areas a full serological analysis should be conducted. The early diagnosis and treatment, following given algorithms as was done in this case, should prevent evolution of the two entities into severe forms which could cause further complications and death. This also highlights the need for a clinician to think about this when examining such patient and to be prepared to act timely with adequate therapy as was done in this case, which apart from drugs required transfusion.

## Data Availability

The original contributions presented in the study are included in the article, further inquiries can be directed to the corresponding author.
